# Private Medical Instruction in Louisville

**Published:** 1847-04

**Authors:** 


### PRIVATE MEDICAL INSTRUCTION IN LOUISVILLE.

Drs. Bayless, Buck and Colescott have just commenced the third annual course of lectures and examinations in which they have given so much satisfaction. We cannot too strongly recommend to students to come to this city and avail themselves of the advantages afforded by this association.

